# Severe Obstructive Sleep Apnea Is Associated with Cholesterol Target Non-Attainment in Patients Undergoing Cardiac Surgery: A Secondary Analysis of a Prospective Observational Cohort

**DOI:** 10.3390/life16101690

**Published:** 2026-10-09

**Authors:** Andrei Raul Manzur, Gabriela Mara, Ana Lascu, Raluca Elisabeta Staicu, Andreea Cristina Chiriiac, Alina Mirela Popa, Loredana Neli Gligor, Andreea-Roxana Florescu, Claudia Borza, Stefan Mihaicuta

**Affiliations:** 1 Doctoral School of Medicine and Pharmacy, Victor Babeș University of Medicine and Pharmacy Timișoara, Piața Eftimie Murgu No. 2, 300041 Timișoara, Romania; andrei.manzur@umft.ro (A.R.M.); alina-mirela.popa@umft.ro (A.M.P.); gligorloredananeli@gmail.com (L.N.G.); andreea.florescu@umft.ro (A.-R.F.); 2 Faculty of Medicine, Vasile Goldiș Western University of Arad, 310414 Arad, Romania; 3 Multidisciplinary Doctoral School, Vasile Goldiș Western University of Arad, 310414 Arad, Romania; 4 Department of Functional Sciences, Discipline of Pathophysiology, Faculty of Medicine, Victor Babeș University of Medicine and Pharmacy Timișoara, Piața Eftimie Murgu No. 2, 300041 Timișoara, Romania; borza.claudia@umft.ro; 5 Centre for Translational Research and Systems Medicine, Victor Babeș University of Medicine and Pharmacy Timișoara, 300041 Timișoara, Romania; 6 Institute for Cardiovascular Diseases Timișoara, Clinic for Cardiovascular Surgery, 300310 Timișoara, Romania; raluca.staicu@umft.ro; 7 Doctoral School of Biomedical Sciences, Faculty of Medicine and Pharmacy, University of Oradea, 410087 Oradea, Romania; chiriiac.andreeacristina@student.uoradea.ro; 8 Department of Medical Disciplines, Faculty of Medicine and Pharmacy, University of Oradea, 410073 Oradea, Romania; 9 Center for Research and Innovation in Precision Medicine of Respiratory Diseases, Department of Pulmonology, Victor Babeș University of Medicine and Pharmacy Timișoara, Piața Eftimie Murgu No. 2, 300041 Timișoara, Romania; stefan.mihaicuta@umft.ro

**Keywords:** obstructive sleep apnea, cholesterol target non-attainment, non-high-density lipoprotein cholesterol, low-density lipoprotein cholesterol, lipid-lowering therapy, cardiac surgery

## Abstract

Background: Obstructive sleep apnea (OSA) has been associated with adverse cardiometabolic profiles, but its relationship with cholesterol target attainment in patients receiving lipid-lowering therapy remains incompletely characterized. We investigated the association between severe OSA and non-attainment of predefined cholesterol thresholds in patients undergoing elective cardiac surgery. Methods: This secondary analysis included 142 adults from a prospectively assembled observational cohort. Participants underwent in-hospital preoperative Type III cardiorespiratory polygraphy, fasting lipid assessment, and documentation of lipid-lowering therapy. Severe OSA was defined as an apnea–hypopnea index ≥30 events/h. The primary outcome was non-high-density lipoprotein cholesterol (non-HDL-C) ≥ 100 mg/dL. Multivariable models were adjusted for age, sex, body mass index, diabetes mellitus, high-intensity statin therapy, and non-statin lipid-lowering therapy. Results: Severe OSA was associated with higher odds of non-HDL-C ≥ 100 mg/dL (adjusted odds ratio [OR] 3.49, 95% confidence interval [CI] 1.56–7.78; *p* = 0.002), LDL-C ≥ 55 mg/dL (OR 2.93, 95% CI 1.18–7.29; *p* = 0.021), and LDL-C ≥ 70 mg/dL (OR 2.30, 95% CI 1.03–5.12; *p* = 0.041). In contrast, associations with continuous LDL-C (*p* = 0.113) and non-HDL-C (*p* = 0.100) concentrations were not statistically significant. No significant associations were observed for triglyceride-related or lipid-derived outcomes. The primary association with non-HDL-C ≥ 100 mg/dL remained significant in the analysis restricted to statin-treated patients. Conclusions: Severe OSA was associated with non-attainment of predefined cholesterol thresholds in this cardiac surgical cohort, with the strongest and most consistent association observed for non-HDL-C ≥ 100 mg/dL. These findings identify an association with categorical cholesterol target non-attainment but do not establish treatment failure, causality, or a generalized increase in cholesterol concentrations.

## 1. Introduction

Despite major advances in lipid-lowering therapy, failure to achieve recommended low-density lipoprotein cholesterol (LDL-C) and non-high-density lipoprotein cholesterol (non-HDL-C) targets remains common among patients at high or very high cardiovascular risk. Achievement of lipid targets is therefore an important component of contemporary cardiovascular risk reduction, particularly in patients with established atherosclerotic cardiovascular disease (ASCVD) [1,2,3,4,5]. However, the clinical relevance of a given lipid threshold depends on the underlying cardiovascular risk profile, and target non-attainment should therefore be interpreted in the context of the population being studied. Identifying clinical characteristics associated with suboptimal cholesterol control may help characterize residual cardiovascular risk and generate hypotheses for improving preventive strategies.

Obstructive sleep apnea (OSA) is highly prevalent among patients with cardiovascular disease and among those referred for cardiac surgery [6,7]. Recurrent upper-airway obstruction produces intermittent hypoxemia and reoxygenation, with downstream activation of sympathetic pathways, oxidative stress, systemic inflammation, and metabolic dysregulation [8,9]. Experimental and clinical evidence further suggests that intermittent hypoxia may influence hepatic lipid metabolism, lipoprotein synthesis and clearance, and pathways involved in cholesterol homeostasis, providing biological plausibility for an association between OSA and adverse lipid profiles. These mechanisms may contribute to the cardiovascular burden associated with OSA, although their clinical relevance in patients already receiving lipid-lowering treatment remains incompletely defined [8,9,10].

Previous studies evaluating the relationship between OSA and lipid metabolism have primarily compared circulating lipid concentrations across OSA categories or investigated dyslipidemia, in general, or untreated populations [11,12,13,14]. Associations between OSA severity and adverse lipid profiles have been reported, but findings have been inconsistent after accounting for obesity and other cardiometabolic factors [13,14]. Moreover, comparatively little is known about lipid target attainment among patients with OSA who are receiving lipid-lowering therapy. Importantly, a lipid concentration above a predefined treatment threshold does not, by itself, establish pharmacological treatment failure because target attainment may also be influenced by treatment intensity, adherence, treatment duration, baseline lipid concentrations, and underlying cardiovascular risk [15]. Accordingly, evaluating target non-attainment rather than treatment failure provides a more cautious framework for examining lipid control in an observational setting.

The present study therefore investigated the association between severe OSA and cholesterol target non-attainment in a prospectively assembled cohort of patients undergoing elective cardiac surgery. Given the observational design, the analysis was intended to identify associations rather than establish a causal effect of OSA on lipid control. We hypothesized that severe OSA would be independently associated with non-attainment of clinically relevant LDL-C and non-HDL-C thresholds after accounting for major measured cardiometabolic characteristics and lipid-lowering treatment intensity. As complementary analyses, we also examined triglyceride concentration, the triglyceride/HDL-C ratio, and the atherogenic index of plasma to characterize whether the observed pattern of associations extended to other lipid-related phenotypes.

## 2. Materials and Methods

### 2.1. Study Design and Ethical Approval

This study represents a secondary analysis of a prospectively assembled observational cohort of adults undergoing elective cardiac surgery at the Institute of Cardiovascular Diseases of Timișoara (Institutul de Boli Cardiovasculare Timișoara, IBCVT), a tertiary referral center in western Romania, between 2022 and 2025. The parent cohort was established to investigate previously unrecognized obstructive sleep apnea (OSA) in patients undergoing cardiac surgery using standardized preoperative cardiorespiratory polygraphy.

The present analysis evaluated the association between severe OSA and cholesterol target attainment in this cardiac surgical cohort. The study was conducted and reported according to the Strengthening the Reporting of Observational Studies in Epidemiology (STROBE) guidelines, and a completed STROBE checklist is provided as Supplementary Material [16].

The study protocol was approved by the Scientific Research Ethics Committee of the Victor Babeș University of Medicine and Pharmacy of Timișoara (approval number 101/19.12.2022; revised in 2025). All procedures complied with the Declaration of Helsinki, and written informed consent was obtained from all participants before enrollment.

### 2.2. Study Population

Consecutive adults scheduled for elective conventional cardiac surgery with cardiopulmonary bypass were prospectively screened for participation. Eligible procedures included isolated coronary artery bypass grafting (CABG), isolated valve surgery, and combined or other complex cardiac procedures. All participants underwent standardized preoperative clinical evaluation, overnight cardiorespiratory polygraphy, fasting lipid assessment, and medication review before surgery.

**Figure 1:**
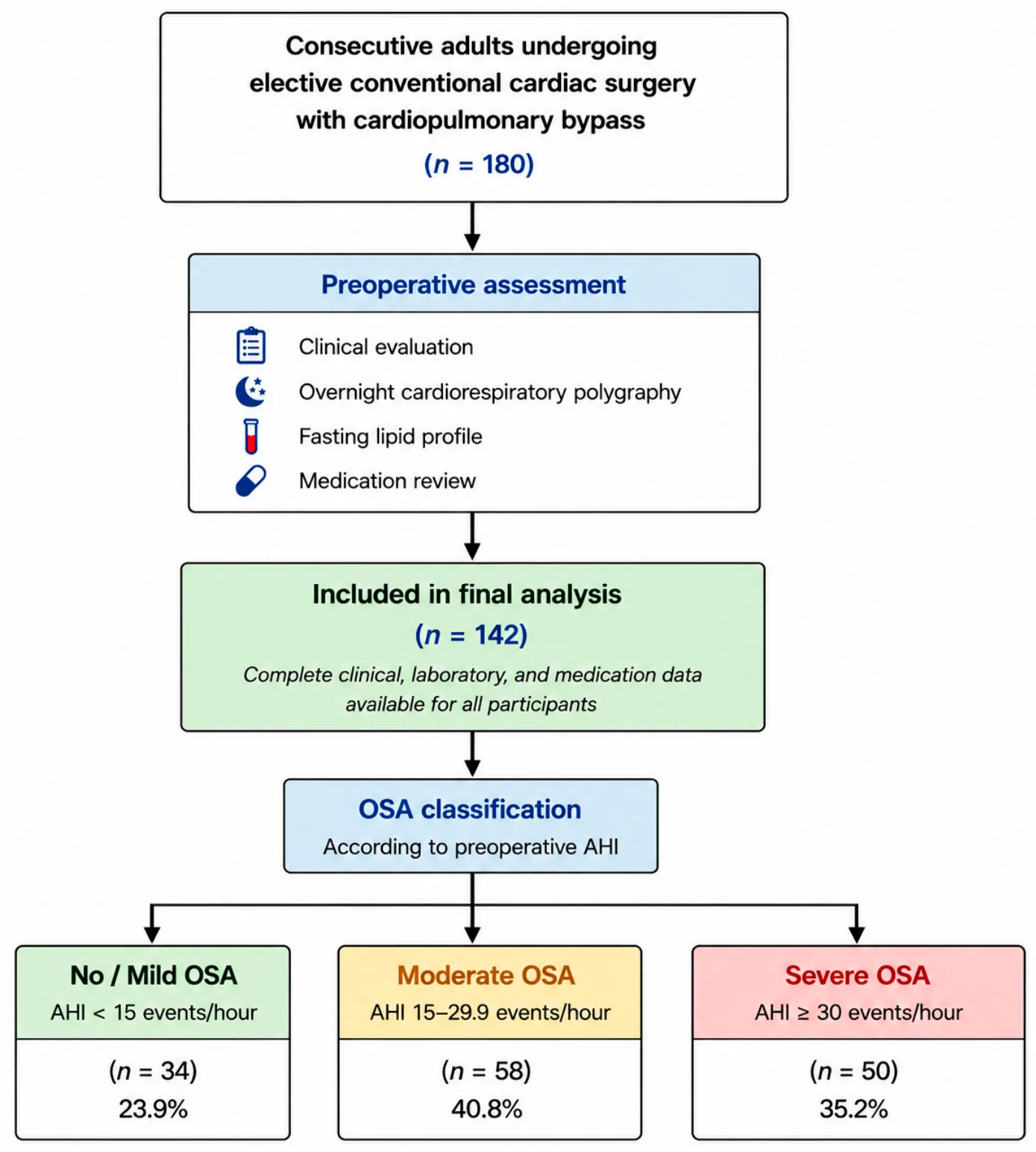
Study flow diagram of patient inclusion and classification according to obstructive sleep apnea severity. Blue indicates preoperative assessment and OSA classification steps; green indicates the final study cohort and no/mild OSA; yellow indicates moderate OSA; and red indicates severe OSA. AHI, apnea–hypopnea index; OSA, obstructive sleep apnea.

An initial cohort of 180 patients scheduled for elective cardiac surgery was considered for the parent study. Of these, 38 did not constitute the final parent-study cohort: 7 withdrew consent, 9 had surgery postponed and initiated OSA therapy, 19 experienced major intraoperative or postoperative cardiovascular complications affecting ventilation-related eligibility for the parent study, and 3 declined surgical intervention. The resulting cohort comprised 142 patients, who constituted the population evaluated in the present secondary analysis (Figure 1).

The cohort included patients undergoing different cardiac surgical procedures and was not restricted to patients with documented atherosclerotic cardiovascular disease (ASCVD). Individual ASCVD status was not systematically recorded for the purposes of the present analysis.

### 2.3. Sleep Study and OSA Classification

All participants underwent overnight type III cardiorespiratory polygraphy in the hospital during the preoperative evaluation, within 24–48 h before surgery, using the Poligraf^®^ VitalNight PG system (Löwenstein Medical, Bad Ems, Germany). Recordings included nasal airflow, thoracoabdominal respiratory effort, pulse oximetry (SpO_2_), pulse rate, and body position. The apnea–hypopnea index (AHI) was automatically generated and subsequently manually reviewed by a trained scorer according to American Academy of Sleep Medicine (AASM) criteria [17]. Technically inadequate recordings were repeated before OSA classification. The parent-study methodology similarly specified automated AHI calculation followed by trained-scorer verification according to AASM criteria.

Patients with predominantly central sleep apnea had been excluded during establishment of the parent cohort before the 180 patients considered for the present analysis. Accordingly, the current cohort was restricted to the obstructive sleep apnea phenotype. None of the included participants had a previously established diagnosis of OSA or was receiving continuous positive airway pressure (CPAP) therapy before the preoperative sleep assessment.

The AHI was calculated as the number of apneas and hypopneas per hour of recording. OSA severity was classified as no/mild OSA (AHI < 15 events/h), moderate OSA (AHI 15–29.9 events/h), and severe OSA (AHI ≥ 30 events/h). Severe OSA served as the primary exposure variable.

For the primary binary analyses, patients with severe OSA (AHI ≥ 30 events/h) were compared with patients without severe OSA (AHI < 30 events/h), who constituted the reference category. Additional robustness analyses evaluated OSA severity using ordinal categories and continuous AHI values.

OSA was diagnosed exclusively for research purposes, and neither participants nor treating physicians were aware of the polygraphy results before surgery. Consequently, perioperative management and pre-existing lipid-lowering treatment were not influenced by knowledge of OSA status.

### 2.4. Clinical Data Collection and Laboratory Measurements

Demographic and clinical characteristics were collected prospectively using standardized case report forms. Baseline variables included age, sex, body mass index (BMI), diabetes mellitus, hypertension, left ventricular ejection fraction, and type of cardiac surgery, categorized as isolated coronary artery bypass grafting, isolated valve surgery, or combined/complex procedures.

Medication history included statin therapy, statin intensity, individual statin agent, and concomitant non-statin lipid-lowering therapy, including ezetimibe and fibrates.

Fasting blood samples were obtained on the morning of surgery before the operative procedure. Serum total cholesterol, LDL-C, HDL-C, and triglycerides were measured using standardized enzymatic assays in the hospital’s accredited clinical laboratory. Non-HDL-C was calculated as total cholesterol minus HDL-C. The triglyceride-to-HDL cholesterol (TG/HDL-C) ratio and the Atherogenic Index of Plasma (AIP) were calculated for exploratory analyses [18,19].

Dietary habits were not systematically assessed. Documented ASCVD status and renal function were not collected as predefined variables for the present analysis.

### 2.5. Lipid-Lowering Therapy

Current lipid-lowering treatment was documented during the preoperative assessment on the basis of patient interview and available medication records. Statin therapy was classified according to the individual agent and treatment intensity based on contemporary European Society of Cardiology recommendations [4]. High-intensity statin therapy was defined as atorvastatin ≥40 mg/day or rosuvastatin ≥20 mg/day; all lower doses were classified as non-high-intensity therapy. Concomitant non-statin lipid-lowering treatment, including ezetimibe and fibrates, was also documented.

Exact duration of lipid-lowering treatment, longitudinal adherence, cumulative treatment exposure, and previous lipid concentrations were not systematically recorded. Accordingly, lipid concentrations above the predefined thresholds were interpreted as target non-attainment at the time of preoperative assessment rather than as evidence of pharmacological treatment failure.

Treatment variables were incorporated into the prespecified multivariable models. Additional sensitivity analyses examined the robustness of the findings after adjustment for statin intensity, statin type, statin dose, and concomitant non-statin lipid-lowering therapy.

### 2.6. Study Outcomes

The primary outcome was non-HDL-C target non-attainment, operationalized as non-HDL-C ≥ 100 mg/dL.

Secondary outcomes comprised LDL-C target non-attainment at thresholds of ≥55 mg/dL and ≥70 mg/dL, together with continuous LDL-C and non-HDL-C concentrations. These thresholds were used as predefined analytic cutoffs and were not assumed to represent individualized guideline-recommended treatment targets for every participant because individual ASCVD status and cardiovascular risk classification were not systematically available [20].

Additional exploratory analyses evaluated triglycerides, the TG/HDL-C ratio, and AIP to characterize whether the observed pattern of associations extended to other lipid-related phenotypes.

Prespecified sensitivity analyses were conducted in statin-treated patients and using alternative treatment-adjustment models. Robustness analyses evaluated OSA severity using three-category ordinal classification and continuous AHI values. Exploratory analyses investigated hierarchical cholesterol target-attainment categories and treatment-intensity phenotypes.

### 2.7. Bias Minimization

Selection bias was minimized through consecutive recruitment using predefined eligibility criteria. Exposure assessment was standardized by applying the same polygraphy protocol and diagnostic criteria to all participants. Because OSA was diagnosed exclusively for research purposes and remained unknown to patients and treating physicians before surgery, lipid-lowering treatment decisions were made independently of OSA status.

Information bias was minimized through prospective collection of demographic, clinical, laboratory, and medication data using standardized case report forms. Potential confounding was addressed through prespecified multivariable adjustment and complementary sensitivity and robustness analyses.

BMI was included as a covariate in the primary multivariable model because of its established relationship with OSA and metabolic risk. Residual confounding from variables not systematically available in the present analysis, including documented ASCVD status, renal function, dietary habits, treatment adherence, and cumulative lipid-lowering treatment exposure, could not be eliminated.

### 2.8. Statistical Analysis

Continuous variables were assessed for normality using visual inspection of histograms and Q-Q plots, supplemented by the Shapiro–Wilk test. Normally distributed variables are presented as means ± standard deviations, whereas non-normally distributed variables are reported as median (interquartile range). Categorical variables are presented as frequencies and percentages.

Baseline characteristics were compared across OSA severity categories using one-way analysis of variance (ANOVA) or the Kruskal–Wallis test for continuous variables and the χ^2^ test or Fisher’s exact test for categorical variables, as appropriate.

The primary analysis evaluated the association between severe OSA (AHI ≥ 30 events/h) and non-HDL-C ≥ 100 mg/dL using multivariable logistic regression, with patients without severe OSA (AHI < 30 events/h) serving as the reference group. The model was adjusted for age, sex, BMI, diabetes mellitus, high-intensity statin therapy, and concomitant non-statin lipid-lowering therapy. Additional multivariable logistic regression models evaluated LDL-C thresholds of ≥55 mg/dL and ≥70 mg/dL, whereas multivariable linear regression was used to evaluate continuous LDL-C and non-HDL-C concentrations.

Sensitivity analyses were performed in statin-treated patients and using alternative adjustment models incorporating statin type, statin dose, ezetimibe therapy, and fibrate therapy. Robustness analyses evaluated OSA severity using ordinal categories and continuous AHI values. Exploratory analyses assessed hierarchical cholesterol target-attainment categories using ordinal logistic regression and treatment-intensity phenotypes using multivariable logistic regression.

BMI was retained in the primary multivariable model irrespective of its univariable association because of its potential confounding relationship with both OSA severity and lipid metabolism. Previously performed obesity-stratified and interaction analyses were used to assess whether the association between severe OSA and the primary lipid endpoint differed according to obesity status.

Categorical and continuous lipid outcomes were considered complementary analyses and were interpreted separately; statistical significance of threshold-based outcomes was not assumed to imply a corresponding significant association across the continuous lipid distribution.

Because complete clinical, laboratory, medication, and sleep study data were available for all participants, complete-case analyses were performed. All analyses were prespecified in a statistical analysis plan finalized before completion of the statistical analyses. Statistical analyses were performed using R (R Foundation for Statistical Computing, Vienna, Austria) and Python (version 3.10). All tests were two-sided, and *p* values < 0.05 were considered statistically significant.

## 3. Results

### 3.1. Study Population and Baseline Characteristics

The present secondary analysis included 142 patients from the final analytic cohort of the parent prospective observational study, with complete demographic, clinical, laboratory, medication, and preoperative cardiorespiratory polygraphy data available for all participants. OSA severity was classified as no/mild OSA (AHI < 15 events/h; n = 34), moderate OSA (AHI 15–29.9 events/h; n = 58), or severe OSA (AHI ≥ 30 events/h; n = 50).

Baseline characteristics are summarized in Table 1. Age, sex, diabetes mellitus, hypertension, left ventricular ejection fraction, type of cardiac surgery, and recorded lipid-lowering treatment did not differ significantly across OSA severity categories. Overall, 122 patients (85.9%) were receiving statin therapy and 40 (28.2%) were receiving additional non-statin lipid-lowering therapy.

BMI was the only measured baseline characteristic that differed significantly across OSA severity categories, increasing from a median of 27.0 (25.0–28.8) kg/m^2^ in the no/mild OSA group to 32.5 (28.0–35.0) kg/m^2^ in the severe OSA group (*p* < 0.001). BMI was therefore included as a prespecified covariate in the multivariable models.

### 3.2. Lipid Profile According to OSA Severity

Lipid parameters according to OSA severity are summarized in Table S1. Overall, conventional lipid parameters and lipid-derived metabolic indices were broadly comparable across the three OSA severity categories. Although median LDL-C and non-HDL-C concentrations were numerically higher in patients with severe OSA than in those with no/mild OSA, none of the unadjusted comparisons reached statistical significance (all *p* > 0.05).

Median LDL-C was 70.0 (45.0–92.3) mg/dL in patients with no/mild OSA and 80.0 (55.0–99.8) mg/dL in those with severe OSA, while median non-HDL-C was 89.9 (66.8–123.2) mg/dL and 104.2 (75.0–128.5) mg/dL, respectively. Total cholesterol and triglyceride concentrations were also numerically higher in the severe OSA group, whereas HDL-C remained relatively stable across OSA severity categories.

Similarly, the TG/HDL-C ratio and AIP did not differ significantly across OSA severity categories.

Given the absence of statistically significant differences in the unadjusted continuous lipid distributions, subsequent multivariable analyses evaluated the prespecified categorical cholesterol thresholds and continuous lipid outcomes after adjustment for predefined covariates.

### 3.3. Severe OSA and Cholesterol Target Non-Attainment

To evaluate the association between severe OSA and cholesterol target non-attainment, multivariable logistic regression analyses were performed after adjustment for age, sex, BMI, diabetes mellitus, high-intensity statin therapy, and non-statin lipid-lowering therapy. Patients without severe OSA (AHI < 30 events/h) served as the reference group. The primary outcome was non-HDL-C ≥ 100 mg/dL.

Overall, non-HDL-C ≥ 100 mg/dL was present in 60 of 142 patients (42.3%), LDL-C ≥ 55 mg/dL in 96 (67.6%), and LDL-C ≥ 70 mg/dL in 72 (50.7%).

**Figure 2:**
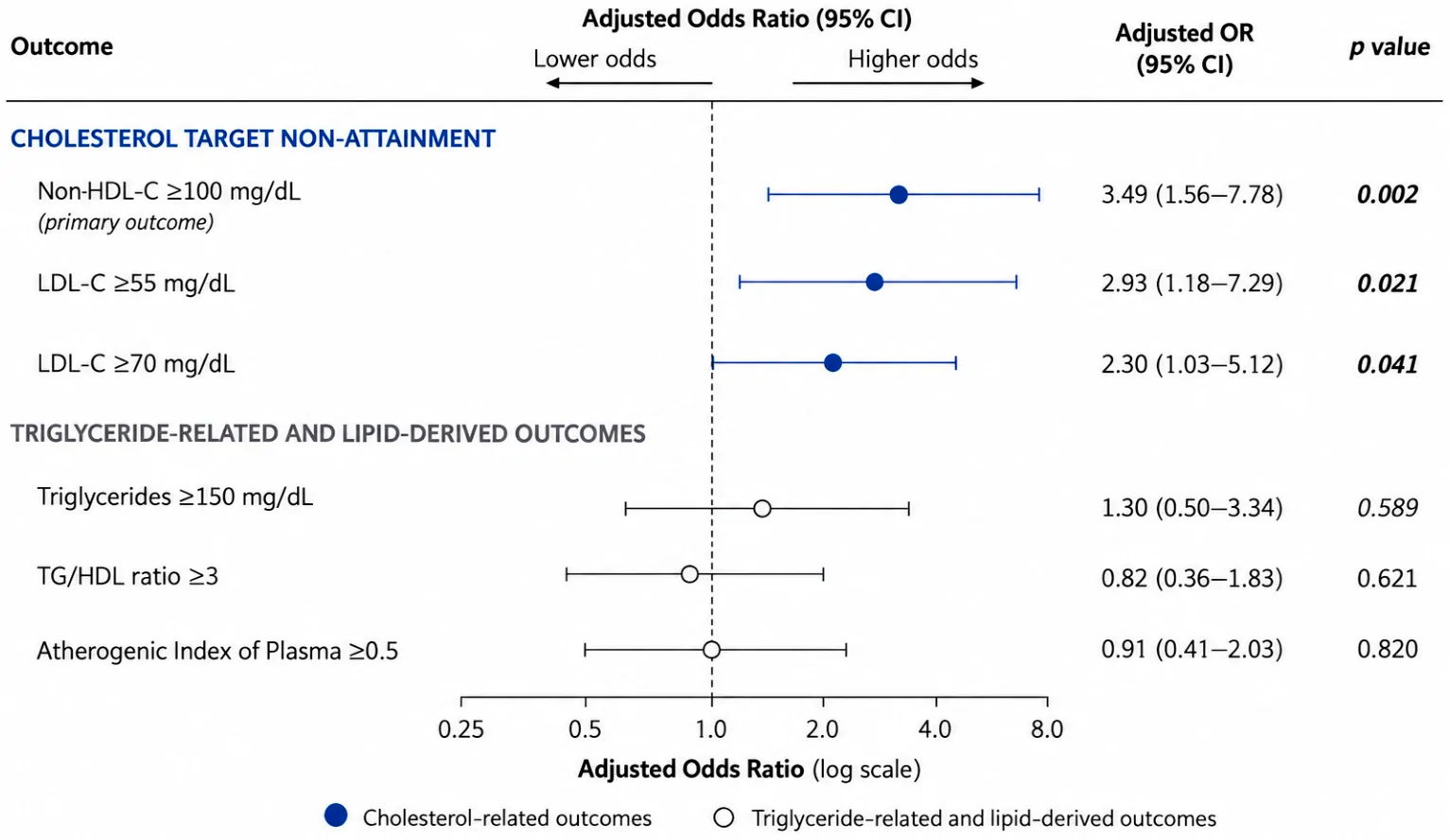
Adjusted associations between severe obstructive sleep apnea and categorical lipid outcomes. Odds ratios (ORs) and 95% confidence intervals (CIs) were derived from multivariable logistic regression models adjusted for age, sex, body mass index, diabetes mellitus, high-intensity statin therapy, and non-statin lipid-lowering therapy. Filled symbols represent cholesterol target non-attainment outcomes, whereas open symbols represent triglyceride-related and lipid-derived outcomes. The vertical dashed line indicates an OR of 1.0.

Severe OSA was associated with higher odds of non-HDL-C ≥ 100 mg/dL (adjusted OR 3.49, 95% CI 1.56–7.78; *p* = 0.002). Associations were also observed for LDL-C ≥ 55 mg/dL (adjusted OR 2.93, 95% CI 1.18–7.29; *p* = 0.021) and LDL-C ≥ 70 mg/dL (adjusted OR 2.30, 95% CI 1.03–5.12; *p* = 0.041) (Table 2 and Figure 2).

In contrast, when cholesterol concentrations were analyzed as continuous outcomes, severe OSA was not significantly associated with either LDL-C (β +9.31 mg/dL, 95% CI −2.19 to 20.81; *p* = 0.113) or non-HDL-C (β +10.91 mg/dL, 95% CI −2.10 to 23.91; *p* = 0.100) after multivariable adjustment.

### 3.4. Associations with Triglyceride-Related and Lipid-Derived Outcomes

Exploratory analyses evaluated whether severe OSA was associated with triglyceride-related and lipid-derived outcomes using the same multivariable adjustment strategy as the primary analysis. No statistically significant associations were observed for TG ≥ 150 mg/dL (adjusted OR 1.30, 95% CI 0.50–3.34; *p* = 0.588), TG/HDL-C ratio ≥ 3 (adjusted OR 0.82, 95% CI 0.36–1.83; *p* = 0.621), or AIP ≥ 0.5 (adjusted OR 0.91, 95% CI 0.41–2.03; *p* = 0.820) (Table 3 and Figure 2).

Similarly, severe OSA was not significantly associated with continuous triglyceride concentrations (β +6.77 mg/dL, 95% CI −14.49 to 28.02; *p* = 0.530), TG/HDL-C ratio (β −0.35, 95% CI −1.91 to 1.21; *p* = 0.659), AIP (β −0.02, 95% CI −0.13 to 0.09; *p* = 0.688), or HDL-C (β +2.53 mg/dL, 95% CI −2.93 to 7.99; *p* = 0.361).

Thus, in these exploratory analyses, the associations observed for the predefined cholesterol thresholds were not accompanied by statistically significant associations with the evaluated triglyceride-related or lipid-derived outcomes.

### 3.5. Sensitivity and Robustness Analyses

In a sensitivity analysis restricted to the 122 patients receiving statin therapy, severe OSA remained associated with non-HDL-C ≥ 100 mg/dL (adjusted OR 3.14, 95% CI 1.35–7.30; *p* = 0.008). Associations with the secondary LDL-C thresholds were attenuated and did not reach statistical significance: adjusted OR 2.52 (95% CI 1.00–6.37; *p* = 0.051) for LDL-C ≥ 55 mg/dL and 1.92 (95% CI 0.84–4.42; *p* = 0.123) for LDL-C ≥ 70 mg/dL.

The association with non-HDL-C ≥100 mg/dL was also similar across alternative treatment-adjustment models incorporating statin use, statin type, statin dose, and individual non-statin therapies, with adjusted ORs for severe OSA ranging from 3.41 to 3.60 (all *p* ≤ 0.004). In the statin-treated subgroup, corresponding estimates ranged from 3.13 to 3.20 (all *p* ≤ 0.008).

Results were further examined using alternative definitions of OSA exposure. In the three-category model, severe OSA was associated with non-HDL-C ≥ 100 mg/dL compared with no/mild OSA (adjusted OR 3.31, 95% CI 1.20–9.12; *p* = 0.021), whereas moderate OSA was not (adjusted OR 0.93, 95% CI 0.37–2.33; *p* = 0.869). When OSA severity was modeled ordinally, each category increase was associated with higher odds of non-HDL-C ≥ 100 mg/dL (adjusted OR 1.92, 95% CI 1.15–3.20; *p* = 0.012). When AHI was modeled continuously, the association did not reach statistical significance (adjusted OR 1.28 per 10-events/h increase, 95% CI 0.99–1.65; *p* = 0.059).

Finally, exploratory analyses stratified by obesity showed associations in the same direction in patients with BMI < 30 kg/m^2^ (adjusted OR 3.58, 95% CI 1.14–11.22; *p* = 0.029) and BMI ≥ 30 kg/m^2^ (adjusted OR 3.22, 95% CI 1.03–10.10; *p* = 0.044). No evidence of interaction between severe OSA and obesity was observed for non-HDL-C ≥ 100 mg/dL (*p* for interaction = 0.883).

### 3.6. Exploratory Analysis of Hierarchical Cholesterol Target Attainment

To further characterize the pattern of cholesterol target attainment, patients were classified into three mutually exclusive hierarchical categories according to the simultaneous achievement of LDL-C < 70 mg/dL and non-HDL-C < 100 mg/dL: concordant target attainment (both thresholds attained), single-threshold non-attainment (either threshold not attained), and concordant target non-attainment (neither threshold attained).

**Figure 3:**
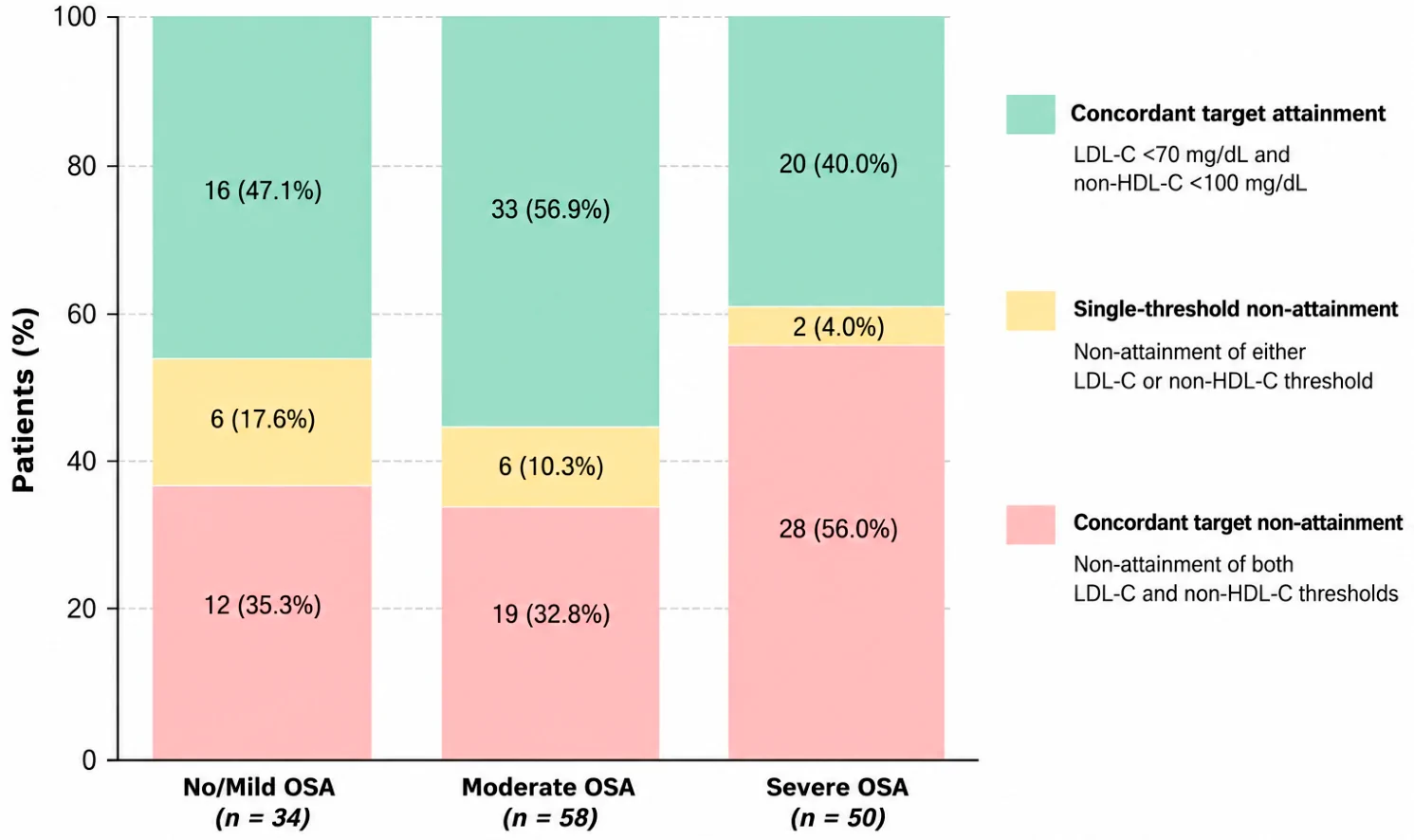
Distribution of hierarchical cholesterol target-attainment categories according to obstructive sleep apnea severity. Patients were classified as having concordant target attainment (LDL-C < 70 mg/dL and non-HDL-C < 100 mg/dL), single-threshold non-attainment (exactly one of the two thresholds not attained), or concordant target non-attainment (LDL-C ≥ 70 mg/dL and non-HDL-C ≥ 100 mg/dL). Bars represent the proportion of patients within each obstructive sleep apnea severity category.

Among patients without severe OSA, 49 of 92 (53.3%) had concordant target attainment, 12 (13.0%) had single-threshold non-attainment, and 31 (33.7%) had concordant target non-attainment. Among patients with severe OSA, the corresponding proportions were 20 of 50 (40.0%), 2 (4.0%), and 28 (56.0%), respectively (Figure 3).

In an ordinal logistic regression model adjusted for age, sex, BMI, diabetes mellitus, high-intensity statin therapy, and non-statin lipid-lowering therapy, severe OSA was associated with higher odds of being in a higher-order target non-attainment category (adjusted OR 2.96, 95% CI 1.36–6.42; *p* = 0.006). In the sensitivity analysis restricted to statin-treated patients, the association remained statistically significant (adjusted OR 2.49, 95% CI 1.11–5.57; *p* = 0.027).

## 4. Discussion

### 4.1. Principal Findings

In this secondary analysis of a prospective observational cohort of patients undergoing elective cardiac surgery, severe OSA was associated with higher odds of non-attainment of predefined non-HDL-C and LDL-C thresholds after adjustment for demographic, clinical, and lipid-lowering treatment variables. The strongest association was observed for the primary outcome of non-HDL-C ≥ 100 mg/dL, for which severe OSA was associated with approximately 3.5-fold higher adjusted odds. Associations were also observed for LDL-C thresholds of ≥55 mg/dL and ≥70 mg/dL.

These categorical findings should be interpreted alongside the continuous analyses, in which adjusted differences in LDL-C and non-HDL-C concentrations were directionally higher in patients with severe OSA but did not reach statistical significance. Thus, the present findings support an association with categorical cholesterol target non-attainment but do not demonstrate a statistically significant difference in continuous cholesterol concentrations.

The primary association with non-HDL-C ≥ 100 mg/dL remained statistically significant in analyses restricted to statin-treated patients and was similar across alternative treatment-adjustment models. Analyses using alternative definitions of OSA exposure also supported the association with severe OSA, although the association with AHI modeled as a continuous exposure did not reach statistical significance.

Exploratory analyses did not identify statistically significant associations between severe OSA and triglyceride-related or lipid-derived outcomes, whereas the hierarchical analysis showed higher odds of belonging to a higher-order cholesterol target non-attainment category among patients with severe OSA. These findings further characterize the observed lipid pattern but should not be interpreted as evidence of biological specificity or resistance to lipid-lowering therapy.

### 4.2. Interpretation of the Primary Findings: Severe OSA and Cholesterol Target Non-Attainment

The principal finding of the present study is an association between severe OSA and non-attainment of predefined cholesterol thresholds rather than evidence that severe OSA directly impairs the response to lipid-lowering therapy. This distinction is important because lipid concentrations were assessed at a single preoperative time point, while treatment adherence, treatment duration, previous lipid concentrations, and cumulative lipid exposure were not systematically available. Accordingly, values above the predefined thresholds should be interpreted as cholesterol target non-attainment rather than pharmacological treatment failure.

The distinction between categorical and continuous outcomes is also relevant to interpretation. Although severe OSA was associated with higher odds of exceeding the predefined non-HDL-C and LDL-C thresholds, adjusted differences in continuous LDL-C and non-HDL-C concentrations did not reach statistical significance. Several factors may contribute to this pattern. Threshold-based analyses characterize the probability of being above a clinically relevant cutoff and may therefore capture differences in the distribution of patients around that cutoff that are not reflected by a statistically significant overall difference in continuous concentrations. Conversely, categorization reduces information contained in continuous measurements and may generate findings that are sensitive to the selected threshold. The present results should therefore not be interpreted as demonstrating a generalized increase in cholesterol concentrations among patients with severe OSA.

The association with the primary outcome was nevertheless supported by several complementary analyses. Severe OSA remained associated with non-HDL-C ≥ 100 mg/dL when the analysis was restricted to statin-treated patients and when alternative approaches to adjustment for lipid-lowering treatment were used. In addition, severe OSA was associated with the primary outcome when OSA was modeled using three severity categories, and increasing OSA severity was associated with higher odds of non-HDL-C target non-attainment in the ordinal model. However, when AHI was analyzed continuously, the association did not reach statistical significance. Taken together, these analyses support the stability of the primary categorical association while also indicating that the relationship should not be interpreted as a simple linear dose–response effect across the full range of AHI.

Importantly, lipid-lowering treatment was common in this cohort, with most patients receiving statins and a smaller proportion receiving additional non-statin therapy. OSA status was determined through preoperative research polygraphy and was not known when lipid-lowering treatment decisions were made. Differences in clinical recognition of OSA are therefore unlikely to explain the observed association. Nevertheless, the observational design and the absence of detailed information on adherence, treatment duration, previous lipid concentrations, and individualized cardiovascular risk prevent determination of whether severe OSA contributes to cholesterol target non-attainment or simply identifies patients with other characteristics associated with poorer cholesterol control.

### 4.3. Triglyceride-Related and Lipid-Derived Outcomes

In contrast to the associations observed for the predefined cholesterol thresholds, severe OSA was not significantly associated with triglyceride concentrations, TG/HDL-C ratio, AIP, or the corresponding categorical triglyceride-related outcomes. These findings indicate that the association observed with cholesterol target non-attainment was not accompanied by statistically significant differences in the triglyceride-related and lipid-derived measures evaluated in this study.

These results should not, however, be interpreted as evidence of biological specificity. Triglyceride concentrations and derived indices such as TG/HDL-C ratio and AIP are influenced by multiple metabolic factors, including adiposity, insulin resistance, glycemic status, dietary factors, and pharmacological treatment. Moreover, absence of a statistically significant association does not establish absence of an underlying relationship, particularly in a cohort of this size. The present analyses therefore provide complementary characterization of the lipid profile associated with severe OSA rather than formal negative-control evidence.

The observed pattern is nevertheless informative when considered alongside the primary findings. Severe OSA was associated with categorical non-HDL-C and LDL-C target non-attainment, whereas no statistically significant associations were identified for the evaluated triglyceride-related outcomes. This pattern does not support a uniformly adverse association between severe OSA and all measured lipid parameters in this cohort. Instead, it highlights the need for further studies specifically designed to determine whether different components of the lipid profile are differentially associated with OSA severity and its underlying physiological disturbances.

### 4.4. Potential Biological Mechanisms

The mechanisms potentially underlying the observed association between severe OSA and cholesterol target non-attainment are likely multifactorial and cannot be established from the present observational study. Nevertheless, several biological pathways provide a plausible framework for interpreting the findings.

Intermittent hypoxemia and reoxygenation, characteristic physiological disturbances of OSA, may promote oxidative stress, systemic inflammation, sympathetic nervous system activation, and metabolic dysregulation. These processes have been linked to alterations in hepatic lipid metabolism, including changes in cholesterol synthesis and lipoprotein clearance, and could therefore contribute to differences in cholesterol homeostasis among patients with severe OSA [21,22,23,24].

Experimental evidence has implicated intermittent hypoxia in pathways affecting lipid metabolism and atherogenesis [25,26], while the low-density lipoprotein receptor (LDLR), 3-hydroxy-3-methylglutaryl-coenzyme A reductase (HMG-CoA reductase), and proprotein convertase subtilisin/kexin type 9 (PCSK9) represent key regulators of cholesterol synthesis and clearance [27,28]. These pathways provide potential mechanistic links between OSA-related physiological disturbances and cholesterol metabolism; however, their specific contribution to the association observed in the present study remains uncertain.

The absence of statistically significant associations with triglyceride-related and lipid-derived outcomes should not be used to infer that these mechanisms selectively affect cholesterol metabolism. OSA-related metabolic disturbances are complex and may affect multiple lipid pathways, while the ability to detect such associations depends on sample size, population characteristics, treatment exposure, and the lipid measures evaluated. Accordingly, the present findings generate hypotheses regarding potential links between severe OSA and cholesterol homeostasis but do not establish a specific biological mechanism or demonstrate that OSA modifies the pharmacological response to lipid-lowering therapy. From a lipid-management perspective, non-HDL-C provides an integrated measure of cholesterol carried by atherogenic lipoproteins and has been proposed as a clinically relevant therapeutic target beyond LDL-C alone [29].

### 4.5. Clinical Implications

The present findings may have clinical relevance for the characterization of cardiovascular risk in patients undergoing cardiac surgery. Severe OSA was associated with higher odds of non-attainment of predefined cholesterol thresholds despite the frequent use of lipid-lowering therapy in this cohort. However, because individualized cardiovascular risk categories were not available for all participants, these thresholds should not be interpreted as guideline-mandated treatment goals for every patient.

Importantly, OSA was identified through preoperative research polygraphy and was not previously diagnosed in the included patients. Consequently, lipid-lowering treatment decisions have been made independently of OSA status. The observed association therefore suggests that severe OSA may identify a subgroup with a greater likelihood of cholesterol target non-attainment, but it does not establish that OSA itself is responsible for inadequate cholesterol control or that treatment of OSA would improve lipid concentrations.

From a clinical perspective, these findings support consideration of OSA as one component of the broader cardiometabolic profile of patients with suboptimal cholesterol control rather than as an indication for a specific change in lipid-lowering management. The present data are insufficient to support routine OSA screening solely on the basis of cholesterol target non-attainment or to recommend treatment intensification based on OSA status. Such decisions should continue to be guided by established cardiovascular risk assessment and lipid-management recommendations.

Future prospective studies should determine whether the association between severe OSA and cholesterol target non-attainment is reproducible in larger and more diverse populations with well-characterized cardiovascular risk, treatment adherence, and longitudinal lipid measurements. Interventional studies would be required to establish whether effective OSA treatment influences cholesterol concentrations, target attainment, or subsequent cardiovascular risk.

### 4.6. Strengths

The present study has several strengths. First, it represents a secondary analysis of a prospectively assembled cohort in which demographic, clinical, laboratory, medication, and sleep-related data were collected using a standardized protocol. OSA was objectively assessed using preoperative cardiorespiratory polygraphy, with automated AHI determination followed by manual review by a trained scorer.

Second, OSA was identified through research polygraphy and was not previously diagnosed in the included patients. Lipid-lowering treatment decisions were therefore made independently of knowledge of OSA status, reducing the possibility that differential clinical recognition of OSA influenced treatment allocation.

Third, the high prevalence of lipid-lowering therapy, with 85.9% of participants receiving statins and 28.2% receiving additional non-statin therapy, allowed the association between severe OSA and cholesterol target non-attainment to be examined in a clinically treated cardiac surgical population. Detailed information on statin intensity, statin type and dose, and adjunctive non-statin therapies also enabled complementary treatment-adjusted and statin-restricted sensitivity analyses.

Finally, the analytical strategy incorporated complementary approaches to both OSA exposure and lipid outcomes, including categorical cholesterol thresholds, continuous lipid concentrations, alternative definitions of OSA severity, obesity-stratified analyses, and hierarchical cholesterol target-attainment categories. This approach allowed the primary association to be evaluated from several perspectives while also identifying important differences between categorical and continuous outcome analyses.

### 4.7. Limitations

Several limitations should be considered when interpreting the present findings. First, the observational design precludes causal inference. Although severe OSA was associated with cholesterol target non-attainment after multivariable adjustment, the present data cannot establish that severe OSA directly contributes to higher cholesterol concentrations, impairs target attainment, or modifies the response to lipid-lowering therapy.

Second, this was a single-center study of patients undergoing elective cardiac surgery, representing a selected cardiovascular population. The cohort included patients undergoing coronary artery bypass grafting, valvular surgery, and complex procedures and was not restricted to individuals with documented atherosclerotic cardiovascular disease. Individual ASCVD status and corresponding cardiovascular risk categories were not systematically available for the present analysis. Consequently, the predefined LDL-C and non-HDL-C thresholds cannot be assumed to represent individualized guideline-recommended treatment targets for every participant, and the generalizability of the findings to other cardiovascular or general populations remains uncertain.

Third, although the multivariable models adjusted for several relevant demographic, clinical, and treatment-related variables, residual and unmeasured confounding cannot be excluded. BMI differed significantly across OSA severity categories and was therefore included in the adjusted models; obesity-stratified analyses did not identify evidence of effect modification. Nevertheless, other potentially relevant factors, including dietary characteristics and renal function, were not available for the present analysis.

Fourth, detailed information regarding adherence to lipid-lowering therapy, duration of treatment, previous lipid concentrations, and cumulative lipid exposure was not systematically available. A single preoperative lipid measurement above a predefined threshold therefore indicates target non-attainment at the time of assessment but cannot establish persistent pharmacological treatment failure or resistance to lipid-lowering therapy.

Fifth, OSA was assessed using in-hospital Type III cardiorespiratory polygraphy rather than attended polysomnography. Because Type III polygraphy does not directly measure sleep stages or total sleep time, AHI may be underestimated when recording time exceeds actual sleep time. In addition, the preoperative hospital environment may have influenced habitual sleep. Although recordings were performed using a standardized protocol, manually reviewed after automated analysis, and technically inadequate recordings were repeated, some degree of OSA misclassification remains possible. Furthermore, oxygen desaturation metrics such as the oxygen desaturation index and time spent below predefined oxygen-saturation thresholds were not available for the present analysis, preventing assessment of whether nocturnal hypoxemic burden was associated with lipid outcomes independently of AHI.

## 5. Conclusions

In this secondary analysis of a prospective observational cohort of patients undergoing elective cardiac surgery, severe OSA was associated with higher odds of non-attainment of predefined non-HDL-C and LDL-C thresholds after adjustment for demographic, clinical, and lipid-lowering treatment variables. The association with the primary non-HDL-C outcome remained evident in several sensitivity analyses, including analyses restricted to statin-treated patients. However, corresponding associations with continuous LDL-C and non-HDL-C concentrations did not reach statistical significance, and no significant associations were observed for the evaluated triglyceride-related or lipid-derived outcomes.

These findings identify severe OSA as a clinical characteristic associated with cholesterol target non-attainment in this selected cardiac surgical population but do not establish causality, pharmacological treatment failure, or a specific effect of OSA on cholesterol metabolism. Further prospective studies incorporating individualized cardiovascular risk, treatment adherence and duration, longitudinal lipid measurements, and detailed measures of nocturnal hypoxemic burden are needed to clarify the nature and potential clinical significance of this association.

## Figures and Tables

**Table 1:** Baseline demographic, clinical, operative, and lipid-lowering treatment characteristics according to obstructive sleep apnea severity. Data are presented as median (interquartile range) for continuous variables and number (percentage) for categorical variables. Continuous variables were compared using the Kruskal–Wallis test. Categorical variables were compared using the χ^2^ test or Fisher’s exact test, as appropriate. Bold text indicates major categories of baseline characteristics. *n*, number of patients; OSA, obstructive sleep apnea; BMI, body mass index; CABG, coronary artery bypass grafting.

Characteristic	Overall (n = 142)	No/Mild OSA (n = 34)	Moderate OSA (n = 58)	Severe OSA (n = 50)	*p* Value
**Demographics**					
Age, years	66.5 (57.2–72.0)	68.0 (55.5–72.8)	67.5 (59.0–71.0)	66.0 (57.2–72.0)	0.978
Male sex, n (%)	102 (71.8)	24 (70.6)	41 (70.7)	37 (74.0)	0.914
BMI, kg/m^2^	29.0 (26.0–33.0)	27.0 (25.0–28.8)	28.5 (25.0–32.0)	32.5 (28.0–35.0)	<0.001
**Clinical characteristics**					
Diabetes mellitus, n (%)	51 (35.9)	13 (38.2)	18 (31.0)	20 (40.0)	0.594
Hypertension, n (%)	129 (90.8)	30 (88.2)	52 (89.7)	47 (94.0)	0.614
Left ventricular ejection fraction, %	50.0 (45.0–50.0)	50.0 (46.2–53.8)	50.0 (45.0–50.0)	50.0 (41.2–50.0)	0.33
**Surgical characteristics**					
CABG	46 (32.4)	16 (47.1)	13 (22.4)	17 (34.0)	
Valve surgery	60 (42.3)	14 (41.2)	26 (44.8)	20 (40.0)	
Complex surgery	36 (25.4)	4 (11.8)	19 (32.8)	13 (26.0)	0.092
**Lipid-lowering therapy**					
Any statin therapy, n (%)	122 (85.9)	29 (85.3)	51 (87.9)	42 (84.0)	0.836
Any non-statin lipid-lowering therapy, n (%)	40 (28.2)	11 (32.4)	14 (24.1)	15 (30.0)	0.656

**Table 2:** Multivariable associations between severe obstructive sleep apnea and cholesterol target non-attainment and continuous cholesterol concentrations. Models were adjusted for age, sex, body mass index, diabetes mellitus, high-intensity statin therapy, and non-statin lipid-lowering therapy. Patients without severe OSA (AHI < 30 events/h) constituted the reference group.

Outcome	Adjusted Estimate (95% CI)	*p* Value
Primary outcome		
Non-HDL-C ≥ 100 mg/dL	OR 3.49 (1.56–7.78)	0.002
Secondary outcomes		
LDL-C ≥ 55 mg/dL	OR 2.93 (1.18–7.29)	0.021
LDL-C ≥ 70 mg/dL	OR 2.30 (1.03–5.12)	0.041
Continuous LDL-C, mg/dL	β +9.31 (−2.19 to 20.81)	0.113
Continuous non-HDL-C, mg/dL	β +10.91 (−2.10 to 23.91)	0.1

**Table 3:** Multivariable associations between severe obstructive sleep apnea and triglyceride-related and lipid-derived outcomes. Models were adjusted for age, sex, body mass index, diabetes mellitus, high-intensity statin therapy, and non-statin lipid-lowering therapy. Patients without severe OSA (AHI <30 events/h) constituted the reference group. Bold text indicates outcome categories. OR, odds ratio; CI, confidence interval; β, regression coefficient; OSA, obstructive sleep apnea; AHI, apnea–hypopnea index; HDL, high-density lipoprotein; TG/HDL, triglyceride-to-high-density lipoprotein cholesterol ratio; AIP, Atherogenic Index of Plasma.

Outcome	Adjusted Estimate (95% CI)	*p* Value
**Categorical outcomes**		
Triglycerides ≥ 150 mg/dL	OR 1.30 (0.50–3.34)	0.588
TG/HDL cholesterol ratio ≥ 3	OR 0.82 (0.36–1.83)	0.621
Atherogenic Index of Plasma ≥ 0.5	OR 0.91 (0.41–2.03)	0.820
**Continuous outcomes**		
Triglycerides, mg/dL	β +6.77 (−14.49 to 28.02)	0.530
HDL cholesterol, mg/dL	β +2.53 (−2.93 to 7.99)	0.361
TG/HDL cholesterol ratio	β −0.35 (−1.91 to 1.21)	0.659
Atherogenic Index of Plasma	β −0.02 (−0.13 to 0.09)	0.688

## Data Availability

The datasets generated and/or analyzed during the current study are available from the corresponding author upon reasonable request, subject to institutional approval and applicable ethical and data protection regulations.

## Supplementary Materials

The following supporting information can be downloaded at: https://www.mdpi.com/article/10.3390/life16101690/s1, Table S1. Conventional lipid parameters and lipid-derived indices stratified by obstructive sleep apnea severity; Table S2. Sensitivity analysis restricted to statin-treated patients; Table S3. Alternative treatment-adjustment models for the primary outcome (residual non-HDL-C ≥ 100 mg/dL); Table S4. Robustness analyses using alternative definitions of obstructive sleep apnea; Table S5. Medication-specific subgroup analyses; Table S6. Stratified and interaction analyses according to statin treatment intensity and obesity; Table S7. Performance of the primary multivariable logistic regression model; Table S8. Exploratory hierarchical cholesterol-control phenotype analyses.

## Abbreviations

The following abbreviations are used in this manuscript:

AASM	American Academy of Sleep Medicine
AHI	Apnea-Hypopnea Index
AIP	Atherogenic Index of Plasma
ANOVA	Analysis of Variance
ASCVD	Atherosclerotic Cardiovascular Disease
BMI	Body Mass Index
CABG	Coronary Artery Bypass Grafting
CI	Confidence Interval
HDL-C	High-Density Lipoprotein Cholesterol
HMG-CoA	3-Hydroxy-3-Methylglutaryl-Coenzyme A
IQR	Interquartile Range
LDL-C	Low-Density Lipoprotein Cholesterol
LDLR	Low-Density Lipoprotein Receptor
non-HDL-C	Non-High-Density Lipoprotein Cholesterol
OR	Odds Ratio
OSA	Obstructive Sleep Apnea
PCSK9	Proprotein Convertase Subtilisin/Kexin Type 9
SAP	Statistical Analysis Plan
SD	Standard Deviation
STROBE	Strengthening the Reporting of Observational Studies in Epidemiology
TG	Triglycerides
TG/HDL-C	Triglyceride-to-High-Density Lipoprotein Cholesterol Ratio
