# Supplementary material for: Severe Obstructive Sleep Apnea Is Associated with Cholesterol Target Non-Attainment in Patients Undergoing Cardiac Surgery: A Secondary Analysis of a Prospective Observational Cohort
Source: Life (Basel). 2026 Oct 9;16(10):1690. doi: 10.3390/life16101690 (PMC13705743; doi:10.3390/life16101690)
Supplement: Supplementary file 1 [file life-16-01690-s001.zip › life-4592015-supplementary.pdf]

Variable	Overall (n = 142)	No/Mild OSA (n = 34)	Moderate OSA (n = 58)	Severe OSA (n = 50)	P value
**Conventional lipid parameters**					
LDL cholesterol, mg/dL	72.0 (49.0–95.0)	70.0 (45.0–92.3)	64.0 (47.9–93.8)	80.0 (55.0–99.8)	0.426
Non-HDL cholesterol, mg/dL	91.4 (71.0–118.6)	89.9 (66.8–123.2)	86.3 (71.0–110.6)	104.2 (75.0–128.5)	0.295
HDL cholesterol, mg/dL	40.4 (30.8–52.8)	38.9 (30.3–51.8)	40.4 (32.0–52.7)	41.6 (29.3–53.4)	0.935
Total cholesterol, mg/dL	135.5 (105.0–161.8)	128.5 (108.8–154.8)	128.0 (104.3–156.8)	154.0 (105.0–171.8)	0.465
Triglycerides, mg/dL	108.0 (84.0–140.0)	103.0 (79.0–149.5)	104.5 (83.5–133.5)	117.5 (94.3–141.5)	0.447
**Lipid-derived indices**					
TG/HDL cholesterol ratio	2.60 (1.90–4.28)	2.36 (1.68–4.17)	2.56 (1.91–4.05)	2.75 (1.91–4.85)	0.546
Atherogenic Index of Plasma (AIP)	0.41 (0.27–0.63)	0.37 (0.23–0.62)	0.41 (0.28–0.61)	0.44 (0.28–0.68)	0.543

Supplementary Table S1. Conventional lipid parameters and lipid-derived indices stratified by obstructive sleep apnea severity. Data are presented as median (interquartile range). Overall comparisons were performed using the Kruskal–Wallis test.

Outcome	Adjusted OR (95% CI)	P value
Residual non-HDL-C ≥100 mg/dL	3.14 (1.35–7.30)	0.008
LDL-C ≥55 mg/dL	2.52 (1.00–6.37)	0.051
LDL-C ≥70 mg/dL	1.92 (0.84–4.42)	0.123

Supplementary Table S2. Sensitivity analysis restricted to statin-treated patients. Models were adjusted for age, sex, body mass index, diabetes mellitus, high-intensity statin therapy, and concomitant non-statin lipid-lowering therapy.

Adjustment model	Adjusted OR (95% CI)	P value
Primary model	3.49 (1.56–7.78)	0.002
Additional adjustment for statin use	3.43 (1.50–7.83)	0.004
Statin type instead of statin intensity	3.41 (1.49–7.79)	0.004
Statin dose instead of statin intensity	3.43 (1.50–7.84)	0.004
Statin type + statin dose + ezetimibe + fibrate	3.49 (1.52–8.03)	0.003
Separate adjustment for ezetimibe and fibrate therapy	3.60 (1.60–8.08)	0.002

Supplementary Table S3. Alternative treatment-adjustment models for the primary outcome (residual non-HDL-C ≥100 mg/dL). All models were adjusted for age, sex, body mass index, diabetes mellitus, and the treatment variables specified above.

OSA exposure definition	Adjusted OR (95% CI)	P value
Moderate OSA (vs no/mild OSA)	0.93 (0.37–2.33)	0.869
Severe OSA (vs no/mild OSA)	3.31 (1.20–9.12)	0.021
Per-category increase in OSA severity	1.92 (1.15–3.20)	0.012

AHI (per 10 events/hour increase)	1.28 (0.99–1.65)	0.059

Outcome: residual non-HDL-C ≥100 mg/dL.

Supplementary Table S4. Robustness analyses using alternative definitions of obstructive sleep apnea. Models were adjusted for age, sex, body mass index, diabetes mellitus, high-intensity statin therapy, and concomitant non-statin lipid-lowering therapy.

**Treatment subgroup**	**Outcome**	**Adjusted OR (95% CI)**	**P value**
**Atorvastatin**	Residual non-HDL-C ≥100 mg/dL	2.78 (1.03–7.51)	0.044
	LDL-C ≥70 mg/dL	1.51 (0.56–4.08)	0.418
	LDL-C ≥55 mg/dL	1.75 (0.61–5.01)	0.297
**Rosuvastatin**	Residual non-HDL-C ≥100 mg/dL	7.54 (1.02–55.52)	0.047
	LDL-C ≥70 mg/dL	6.44 (0.83–49.68)	0.074
	LDL-C ≥55 mg/dL	8.04 (0.96–67.25)	0.054
**No ezetimibe therapy**	Residual non-HDL-C ≥100 mg/dL	3.24 (1.29–8.11)	0.012

Supplementary Table S5. Medication-specific subgroup analyses. Models were adjusted for age, sex, body mass index, diabetes mellitus, statin dose (where applicable), and concomitant lipid-lowering therapy. Results should be interpreted cautiously because of the limited sample size within several treatment subgroups.

A. Stratified analyses

**Stratum**	**Outcome**	**Adjusted OR (95% CI)**	**P value**
Non-high-intensity statin therapy	Residual non-HDL-C ≥100 mg/dL	4.86 (1.44–16.36)	0.011
	LDL-C ≥70 mg/dL	3.69 (1.09–12.48)	0.035
	LDL-C ≥55 mg/dL	4.14 (1.12–15.24)	0.033
High-intensity statin therapy	Residual non-HDL-C ≥100 mg/dL	2.74 (0.82–9.14)	0.1
	LDL-C ≥70 mg/dL	1.24 (0.38–4.01)	0.723
	LDL-C ≥55 mg/dL	1.95 (0.50–7.65)	0.339

BMI <30 kg/m^2	Residual non-HDL-C ≥100 mg/dL	3.58 (1.14–11.22)	0.029
BMI ≥30 kg/m^2	Residual non-HDL-C ≥100 mg/dL	3.22 (1.03–10.10)	0.044
	LDL-C ≥70 mg/dL	2.91 (1.01–8.42)	0.048

B. Interaction analyses

**Interaction term**	**Outcome**	**Interaction P value**
Severe OSA × High-intensity statin therapy	Residual non-HDL-C ≥100 mg/dL	0.786
	LDL-C ≥70 mg/dL	0.275
	LDL-C ≥55 mg/dL	0.704
Severe OSA × Obesity	Residual non-HDL-C ≥100 mg/dL	0.883
	LDL-C ≥70 mg/dL	0.947
	LDL-C ≥55 mg/dL	0.812

Supplementary Table S6. Stratified and interaction analyses according to statin treatment intensity and obesity. All models were adjusted using the same covariates as the primary analysis, excluding the stratification variable where appropriate. No statistically significant interactions were observed.

**Diagnostic**	**Result**	**Interpretation**
Area under the receiver operating characteristic curve (AUC)	0.663	Modest discrimination
Hosmer–Lemeshow goodness-of-fit test	P = 0.891	No evidence of poor calibration
Brier score	0.224	Acceptable overall prediction error
Variance inflation factor (range)	1.04–1.23	No evidence of multicollinearity

Supplementary Table S7. Performance of the primary multivariable logistic regression model.

Analysis	Adjusted OR (95% CI)	P value
**Primary cohort**		
Hierarchical cholesterol-control phenotype (ordinal model)	2.96 (1.36–6.42)	0.006
**Statin-treated cohort**		
Hierarchical cholesterol-control phenotype (ordinal model)	2.49 (1.11–5.57)	0.027

Analysis	Adjusted OR (95% CI)	P value
**Primary cohort**		
Hierarchical cholesterol-control phenotype (ordinal model)	2.96 (1.36–6.42)	0.006
**Statin-treated cohort**		
Hierarchical cholesterol-control phenotype (ordinal model)	2.49 (1.11–5.57)	0.027

Supplementary Table S8. Exploratory hierarchical cholesterol-control phenotype analyses. Ordinal logistic regression models evaluated progression toward increasingly adverse hierarchical cholesterol-control phenotypes (optimal cholesterol control, partial cholesterol treatment-goal failure, and concordant cholesterol treatment-goal failure). Models were adjusted for age, sex, body mass index, diabetes mellitus, high-intensity statin therapy, and non-statin lipid-lowering therapy. In the statin-treated sensitivity analysis, adjustment variables were identical except that all participants were receiving chronic statin therapy.
